# Deep penetrating nevus: a case report and brief literature review

**DOI:** 10.1186/1746-1596-1-31

**Published:** 2006-09-25

**Authors:** Victor S Flauta, Daniel C Lingamfelter, Linh M Dang, Kamani M Lankachandra

**Affiliations:** 1 Department of Pathology, University of Missouri-Kansas City School of Medicine and Truman Medical Centers, Kansas City, Missouri, USA; 2 Department of Pathology, Baylor College of Medicine, Houston, Texas, USA

## Abstract

**Background -:**

Deep penetrating nevus (DPN) is a distinct variant of melanocytic nevus and remains a histopathologic challenge to pathologists because of its resemblance to blue nevus, malignant melanoma, pigmented Spitz nevus, and congenital melanocytic nevus. It often goes unrecognized due to its relative rarity.

**Case presentation -:**

Here we report a case of DPN of the left anterior leg in a 51-year old female. A brief review of the literature shows that these lesions have a distinct growth pattern and cellular morphology that can differentiate these lesions from other entities including malignant melanoma.

**Conclusion -:**

It is important to recognize these features because DPN carries a better prognosis than malignant melanoma.

## Background

Deep penetrating nevus is a distinct variant of melanocytic nevus. It is characterized by its dark pigmentation, occurring mostly on the face, neck, or shoulder, and usually measures less than 1 cm in diameter [1]. Due to its striking histologic similarities with other cutaneous pigmented lesions, it is often difficult to recognize as a separate entity and is consequently misinterpreted as malignant melanoma [2]. Here, we present a case of DPN, describe its histopathologic and clinical features, and provide a brief review of the literature.

## Case presentation

A 51-year old Caucasian female presented to an outside institution in 1997 with a left shin lesion and was diagnosed with melanoma. A wide excision was performed with two subsequent re-excisions performed in 2001. At that time she received a split-thickness skin graft and a left inguinal sentinel node biopsy which was negative for malignancy. Punch biopsies were also obtained from multiple tan-brown lesions on both legs. These biopsies showed both typical and atypical melanocytic hyperplasia along with compound and dermal nevi. In 2002, more biopsies of the left lower leg were performed, revealing squamous cell hyperplasia. In February of 2005, she presented to us with a left lower leg skin lesion in the same area as the prior melanoma. The lesion was excised and submitted for pathologic evaluation.

### Pathologic description of the lesion

The specimen was a skin ellipse measuring 1.5 × 0.7 cm with an excisional depth of 0.7 cm. Centrally located on the external surface of the skin was a dark brown, discolored macular area with irregular borders measuring 0.1 × 0.1 cm. The lesion did not appear to involve the margins of the specimen, which was entirely submitted for formalin processing.

**Figure 1:**
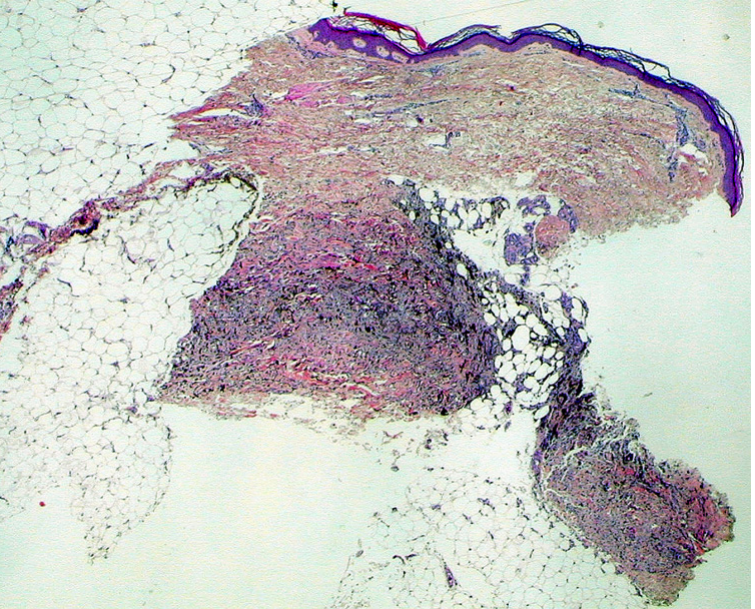
**(H&E, ×20): **Nested proliferations of spindle and epithelioid cells are seen within the deep reticular dermis and subcutaneous tissue and are arranged in a wedge shape with a broad base in parallel with the dermis and an apex directed toward the epidermis.

**Figure 2:**
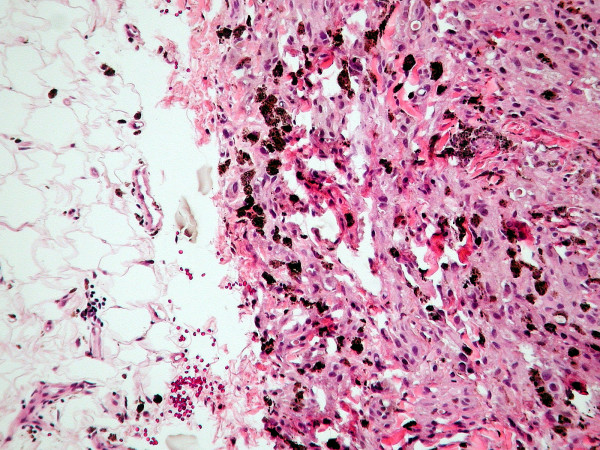
**(H&E, ×100): **Nests of spindle cells and epithelioid-like cells diffusely infiltrate the surrounding tissues, including the deep subcutaneous adipose tissue on the left. Note the abundance of melanophages.

**Figure 3:**
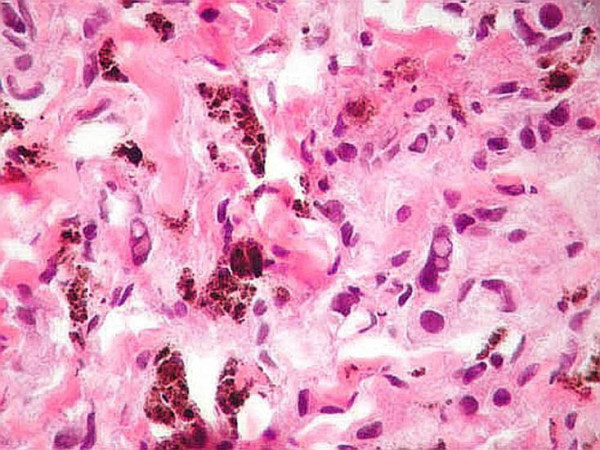
**(H&E, ×400): **The tumor cells reveal severe cytologic atypia and pleomorphism. Several cells display nuclear vacuolation.

**Figure 4:**
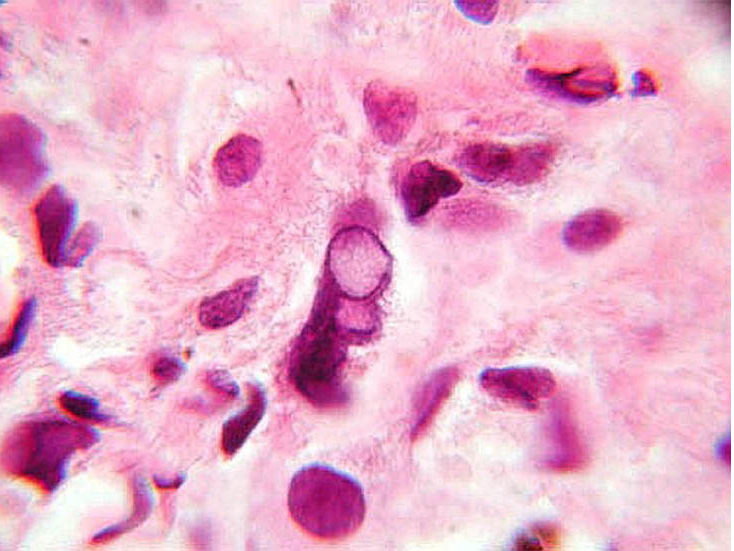
**(H&E, ×1000): **The tumor cell in the center displays a large, pleomorphic nucleus containing several vacuoles.

**Figure 5:**
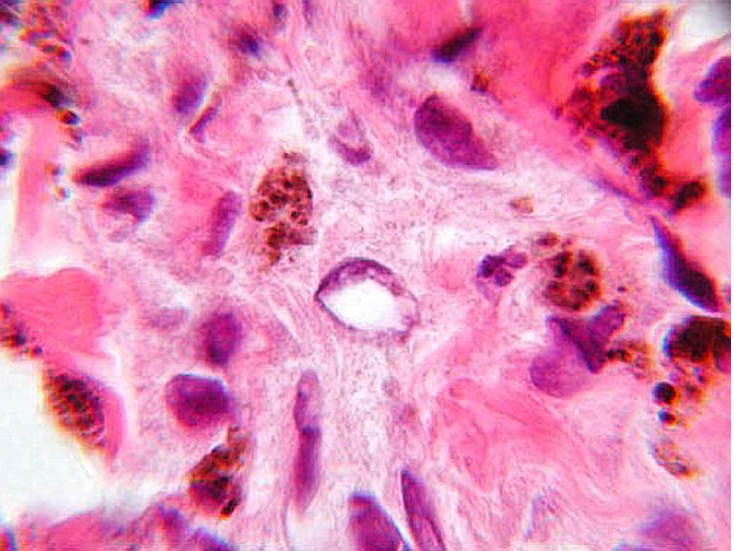
**(H&E, ×1000): **The nucleus of this tumor cell displayed in the center of the field reveals irregularly marginated chromatin with a pale, central pseudoinclusion.

5-um sections from the formalin-fixed, paraffin-embedded tissue were used for routine light microscopic study. The sections showed deep dermal and subcutaneous nested proliferations of spindle and epithelioid cells arranged in a wedge-shaped pattern with the base in parallel with the reticular dermis andthe apex oriented toward the epidermis (Fig. 1). These nests diffusely penetrated and infiltrated the surrounding tissues (Figure 2). A junctional component was present, but scant. Abundant melanophages along with some pigment incontinence were present. The tumor cells were characterized by severe cytologic atypia and pleomorphism (Figure 3) with occasional nuclear vacuoles and large pseudoinclusions (Figures 4, 5). The lesion was subsequently diagnosed as a deep penetrating nevus.

## Discussion

DPN was first described by Seab in 1989. Since then, 169 cases have been reported in the English literature [see Additional file 1]. This lesion can affect a wide variety of age groups but has a predilection for those in their twenties. The size can range from 2 mm to 1 cm. The lesion may go undiagnosed from a few weeks up to eight years. Although it appears to have a predilection for the face, neck and shoulder, it can also be located in other parts of the body including the head, upper and lower extremities, trunk, shoulder, and abdomen.

Clinically, DPN usually presents as a darkly-pigmented papule or nodule that generally appears dark, ranging from pink to black in color [2-6]. The initial clinical diagnosis often can be misleading, as DPNs have been initially misinterpreted as a number of different entities including malignant melanoma, possible melanoma, nevus, blue nevus, seborrheic keratosis, dysplastic nevus, tattoo, hemangioma, cyst, enlarging mole, and pigmented Spitz.

DPN has distinct morphologic and histologic features. It is usually characterized by a sharply-demarcated, wedge-shaped lesion with its base in parallel with the epidermis andits apex oriented toward the subcutaneous fat or deep reticular dermis [1,3-6]. Oddly enough, this case revealed an exactly opposite architecture, with a deep base and an apex pointing toward the epidermis. DPN is composed of loosely arranged nests or fascicles of deeply pigmented nevus cells admixed with melanophages scattered throughout the dermis, even extending into the subcutaneous fat [1-6]. It is usually found closely associated with dermal appendages, such as hair follicles and sweat glands, as well as with blood vessels and nerves [1-3,6]. Some inconspicuous nests or fascicles can be found at the dermoepidermal junction [1,2,5]. In contrast, malignant melanoma starts at the dermoepidermal junction and pierces through the dermis in an irregular and destructive pattern [2].

At a higher magnification, this lesion demonstrates clustering of pigmented nevus cells with pleomorphic, vacuolated nuclei with smudged chromatin, rare or no mitoses, and scattered melanophages. Nuclear vacuoles and large pseudoinclusions are often observed [1-3,5]. If an inflammatory reaction is seen, it is usually composed of small lymphocytes [2,4,5]. Some lesions display a low-grade cytologic atypia with variable nuclear size, shape, and hyperchromacity [1,3,4].

Even while DPN displays such characteristics as a unique architectural pattern and a minimal mitotic rate, such characteristics as cellular atypia, pleomorphism and an infiltrating growth pattern leave little to wonder as to why DPN can be so difficult to differentiate from malignant melanoma. However, Ball and Golitz provide a comprehensive, tabulated review of the differences between malignant melanoma and DPN [7].

Barnhill described a case of plexiform spindle cell nevus as a plexiform pattern of fascicles composed of spindle cells extending through the reticular dermis. It was also intimately associated with adnexal structures, nerves and blood vessels with intervening normal dermis. Since these features are similar to DPN, the plexiform spindle cell nevus has now been considered as DPN [3].

Immunohistochemical analysis usually shows positivity for both S-100 and HMB-45 while stains for keratin are universally negative [2], thereby providing no value in differentiating DPN from malignant melanoma and leading to the authors' decision not to perform immunohistochemistry on this case. An immunoperoxidase technique with the ability to differentiate these two entities has not been identified as far as the authors are aware and provides an opportunity for further diagnostic advancement.

Excision has been the unanimous choice of treatment [2-6]. After its removal, no further treatment is recommended as long as it is completely excised [5]. Metastasis has never been observed in this lesion, hence resulting in a favorable prognosis in contrast to malignant melanoma.

Of all the reported cases, there were no recurrences after follow-ups of one to twenty-three years other than that featured in a report by Robson in 2003 [6], in which one lesion out of 31 cases recurred after one year. However, after re-excision this lesion did not recur thereafter. The patient we present is unique in that numerous neoplasms were diagnosed in the same area of the left lower leg and ranged from such benign entities as nevi up to the malignant process of malignant melanoma. The fact that the specimen from which we diagnosed DPN was derived from the same location as the previous melanoma, diagnosed elsewhere, begs the question as to whether this supposed melanoma was actually a deep penetrating nevus that recurred.

## Conclusion

Deep penetrating nevus is a distinct entity that can mimic various other skin lesions, most notably malignant melanoma. However, distinct architectural and cytologic characteristics should allow the pathologist to arrive at the correct diagnosis. These lesions have shown zero metastatic potential, and recurrences are exceptional. Therefore, accurately diagnosing this neoplasm is important because of its favorable prognosis and less burdensome treatment strategy, in stark contrast to that of malignant melanoma. Patients may thereby avoid unnecessary, often grueling, treatment courses as well as the emotional and psychological impact a misdiagnosis can have on them and their families.

## Supplementary Material

Additional File 1 Table 1: Reported Cases of Deep Penetrating Nevus. Herein we detail the existing reported cases of deep penetrating nevus including the literature source, demographics, clinical presentation, histopathology, treatment, and follow-up. Click here for file
