# Supplementary material for: Deep penetrating nevus: a case report and brief literature review
Source: Diagn Pathol. 2006 Sep 25;1:31. doi: 10.1186/1746-1596-1-31 (PMC1586213; doi:10.1186/1746-1596-1-31)
Supplement: Additional File 1 — Table 1: Reported Cases of Deep Penetrating Nevus. Herein we detail the existing reported cases of deep penetrating nevus including the literature source, demographics, clinical presentation, histopathology, treatment, and follow-up. [file 1746-1596-1-31-S1.doc]

**Table 1: Reported Cases of Deep Penetrating Nevus**

| Literature  source | No. of  pts | Age/Sex | Size | Duration | Location | Clinical Presentation | Microscopic findings | Initial Clinical Dx | Tx | Follow-up |
| --- | --- | --- | --- | --- | --- | --- | --- | --- | --- | --- |
| Seab^2^, 1989 | 70 | 3-63 (most 10-30)  53%F, 47%M | 2-9mm diameter | Not avail | Head, face neck (29)  Upper arm, shoulder (15) | Darkly pigmented papule or nodule | Lesions had loosely arranged nests/fascicles of pigmented nevus cells with melanophages. They were usually surrounded by hair follicles, sweat glands, BV, and nerves. There was little to no inflam. rxn, and were usually at the dermoepidermal jxn. Lesions had hyperchromatic nuclei, but chromatin was smudged and structureless. There were nuclear vacuoles and large pseudoinclusions. Mitoses were rare or absent. Lesions had very little stromal rxn. | Malignant melanoma (29%) | Simple excision | F/U on 48 pts ranged from 1-23 yrs with mean F/U of 7 yrs; no recurrences or metastasis |
| Cooper^5^, 1992 | 41 | 3-47 (X=24.9)  26F, 15M | All but 2 were 6mm or less; other 2 were 1 cm diameter | “recent” to 8 years | Face(13)  Upper Ext (11)  Lower Ext (6)  Trunk (11) | Slightly elevated dome-shaped papule; color ranged from brown-black to pink | Most had a wedge shape and penetrated deep reticular dermis. Lesions had relatively confluent growth centrally and fascicular-plexiform pattern at periphery. Most had jxnal melanocytes, and nuclear pseudoinclusions were observed in one-half of lesions. Most had abundant interstitial melanophages. Lesions had scattered, focal, and patchy infiltration of small lymphocytes. | Nevus (20)  ? Malignant melanoma (18)  Blue Nevus (9)  Seborrheic keratosis (2)  Dysplastic nevus (1)  Tattoo (1)  Hemangioma (1)  Cyst (1) | Excision | F/U of 3 months; no recurrence |
| Barnhill^3^, 1991 | 12 | 9-42 (X=22.5)  7F, 5M | unavail | unavail | Head/Neck (2)  Shoulder (4)  Back (3)  Chest (1)  Arm (1)  Abd (1) | Slightly elevated and bluish or almost black papule | Arch. was symmetrical, wedge-shaped, and had plexiform configuration  Lesions had perineural involvement and most infiltrated arrector pili muscle. Most had jxnal nesting of spindle cells and involved papillary dermis and had irregular nuclear contour and some hyperchromasia. Some had intranuclear cytoplasmic vacuoles, and mitotic figures were rare. There were variable numbers of melanophages found with spindle cell aggregates. | Blue nevus,  Atypical nevus,  Melanoma | Excision | Not Available |
| Mehregn^4^1993 | 14 | 17-35 (X=26)  8F, 7M | unavail | 3 months-35 yrs | Face (6 total; 4 cheek, 1 lip, 1 neck)  Upper arm (5)  Leg (1)  Breast (1) | Not avail | Lesions had wedge-shape, extending from upper dermis into narrow portion that penetrated subcutaneous fat. Deep nests had spindle-shaped cells in fascicles/nests of melanocytes with abundant cytoplasm and dustlike pigment granules. There was low-grade inflam cell rxn, and mitotic figures were rare. Melanophages were seen in the deep part of the lesion. There was some cytologic atypia with variety in size and shape and hyperchromacity of nuclei in the deeper portions. | Not avail | Excision | No recurrence or metastasis over 6 month to 8 yr period |
| Robson^6^, 2003 | 31 | 3-56 (X=25.8)  17F, 14M | 2-10mm diameter | Few weeks-2+yrs | Face/neck (10)  Back (6)  Lower Ext (7)  Abd (1)  3 unknown | Darkly pigmented papule/nodule with none to mild epidermal changes | Lesions had wedge-shaped growth with the base towards the epidermis and apex extending into subcutaneous fat or reticular dermis. Deeper portions followed dermal appendages or BV. Cells were arranged in loose nest/fascicles oriented vertically. Fascicles had plump fusiform to spindle cells. | Nevus, enlarging mole, blue nevus, melanoma, pigmented spitz | Excision | 1 lesion recurred after 1 yr but no recurrence afterward; all other lesions had no recurrence or metastasis in 1-17 yr f/u |
| Hassan^1^, 2003 | 1 | 25 | 2-8mm diameter | 7 yrs | Multiple R periauricular | 7 slightly elevated, smooth-surfaced, well-defined, darkly pigmented lesions in a linear distribution. One lesion was surrounded by 3 tiny satellite lesions | Lesions were symmetrical, well-demarcated, and wedge-shaped with apex in subcutaneous fat. Melanocytes were arranged in nests/fascicles, and lesions were found around hair follicles, sweat glands, BV, and nerves. Most melanocytes had monomorphous nuclei. Some had low-grade cytologic atypia with variable nuclear size, shape and hyperchromasia. There were no mitotic figures. Some nests were at the dermoepidermal jxn. | Deep penetrating nevus | Excision | Not Available |
| Current Case, 2005 | 1 | 51/F | 1 mm | Uncertain | Left leg | Dark pigmented papule | Lesion showed a deep dermal and subcutaneous proliferation of spindle and epithelioid cells with deep penetrating and infiltrating growth pattern. The tumor cells were characterized by severe cytologic atypia, pleomorphism and mitotic activity | Deep penetrating nevus | Excision | Not available |

Legend: M: male, F: female, No.: number, pts: patients, avail: available, unavail: unavailable, Ext: extremity, X: mean, R: right, L: left, BV: blood vessel, F/U: follow-up, yr: year, Abd: abdomen, ?: possible, Dx: diagnosis, inflam: inflammation, rxn: reaction, jxn: junction, pos: positive, neg: negative, Tx: treatment, Arch: architecture
